# Biased pollen transfer by bumblebees favors the paternity of virus-infected plants in cross-pollination

**DOI:** 10.1016/j.isci.2023.106116

**Published:** 2023-02-24

**Authors:** Alex M. Murphy, Sanjie Jiang, James A.D. Elderfield, Adrienne E. Pate, Chay Halliwell, Beverley J. Glover, Nik J. Cunniffe, John P. Carr

**Affiliations:** 1 Department of Plant Sciences, University of Cambridge, Downing Street, Cambridge, CB2 3EA United Kingdom

**Keywords:** Interaction of plants with organisms, Plant ecology, Plant pathology, Plant products

## Abstract

We used a *green fluorescent protein* marker gene for paternity analysis to determine if virus infection affected male reproductive success of tomato in bumblebee-mediated cross-pollination under glasshouse conditions. We found that bumblebees that visited flowers of infected plants showed a strong preference to subsequently visit flowers of non-infected plants. The behavior of the bumblebees to move toward non-infected plants after pollinating virus-infected plants appears to explain the paternity data, which demonstrate a statistically significant ∼10-fold bias for fertilization of non-infected plants with pollen from infected parents. Thus, in the presence of bumblebee pollinators, CMV-infected plants exhibit enhanced male reproductive success.

## Highlights

• Bees preferentially transfer virus-infected plant pollen to non-infected plants
• Paternity analysis shows this increases gene flow from virus-infected plants
• Enhanced gene flow from susceptible plants may counteract selection for resistance

## Graphical abstract

**Figure:**
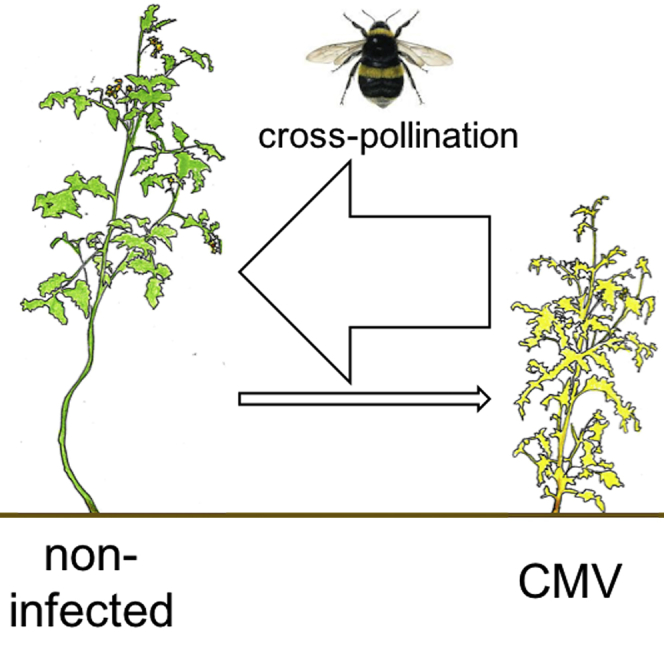

## Introduction

Cross-pollination, the transfer of pollen grains from the stamens of one plant to the stigma of another, has the potential to increase genetic diversity of the next generation.^1^ Cross-pollination also contributes to gene flow between and within populations. Paradoxically, whilst gene flow can be an important agent of evolution, for example, by facilitating dissemination of a new trait, it can also act as an ‘evolutionary glue’ that limits genetic diversity within a species, preventing genetic drift.^2^^,^^3^^,^^4^^,^^5^ A fuller understanding of the mechanisms shaping pollination is important, not only because of its role in gene flow, but also for practical reasons, particularly in the light of global pollinator decline.^6^ Over three-quarters of major crops grown worldwide and 90% of wild flowering plants rely on animal-mediated pollination to set fruit and seed and, out of all animals, bees are the principal pollinators.^7^^,^^8^^,^^9^ Much research has been devoted to understanding the inherent features of flowers that influence pollinator behavior (e.g., floral signals and rewards^1^ or the trade-offs between attracting pollinators and deterring predation.^5^ However, the effects of plant pathogen infection on pollen movement between plants have not been deeply explored.

It was previously shown that volatile organic compounds emitted by virus-infected bean (*Phaseolus vulgaris* L.) and tomato (*Solanum lycopersicum* L.) plants attract bumblebees (*Bombus terrestris*).^10^^,^^11^ When bumblebees visit tomato flowers, they vibrate to extract pollen (‘buzz pollination’) and, in doing so, maximize seed production by increasing the self-pollination rate.^11^ Cucumber mosaic virus (CMV) infection decreases tomato seed yield, except in the presence of bumblebees whose buzz pollination activity rescues infected plant seed yields to levels equivalent to those of non-infected plants.^11^ It was concluded that a greater preference of pollinators for flowers of virus-infected plants would increase their success as female parents, by increasing the probability of ovule fertilization.^11^ In addition, it was inferred that for a wild population of plants evolving and that this could constitute a ‘payback’ from a virus to its susceptible hosts that would blunt selection pressure for resistance and would, as a byproduct, enable survival of alleles for susceptibility within a plant population.^11^

In this study, we used a transgene encoding the green fluorescent protein (GFP) as a marker gene for paternity analysis to determine if CMV infection affected male reproductive success in bumblebee-mediated cross-pollination of tomato under glasshouse conditions. We found that bumblebees that first visited flowers of infected plants showed a strong preference to subsequently visit flowers of non-infected plants; suggesting the insects either find infected plant pollen distasteful, or direct contact with infected flowers repellent. The behavior of the bumblebees, i.e., to move toward non-infected plants after pollinating the infected, appears to explain the paternity data, which show a statistically significant ∼10-fold bias for fertilization of non-infected plants with pollen from infected parents. Thus, in the presence of bumblebee pollinators, CMV-infected plants exhibit enhanced male and female reproductive success.

## Results and discussion

### Establishment of a paternity tracking system

**Figure 1:**
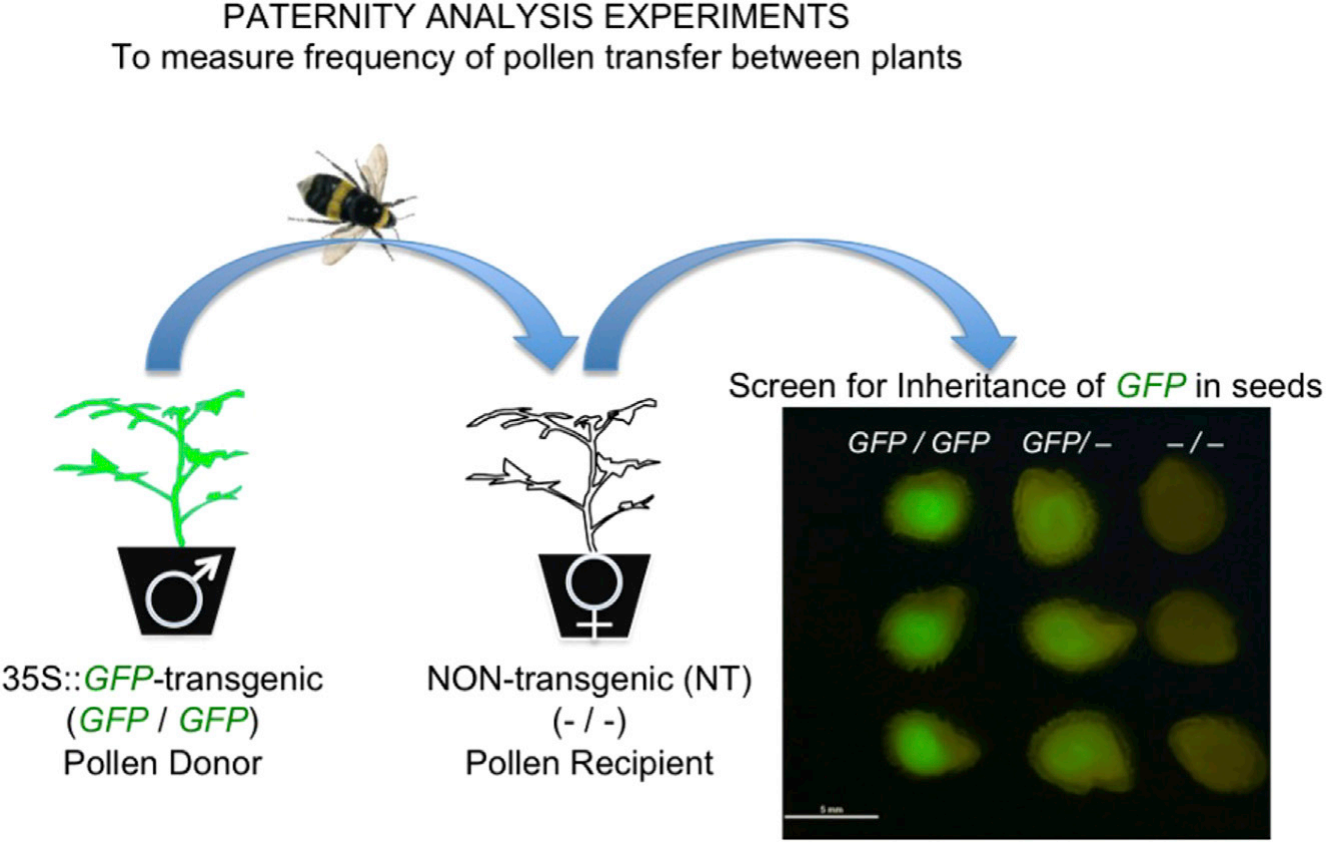
Experimental design for measurement of cross-pollination rates Pollen from donor transgenic tomato constitutively expressing green fluorescent protein (35S:GFP) provides a visually scorable trait in offspring from cross-pollinated non-transgenic (NT) pollen-recipient plants. This allows an estimation of cross-pollination rates between plants that are genetically identical except for the possession of the *GFP* gene. The effect of virus infection on the rate of cross-pollination can be examined by inoculating either the 35S:GFP expressing or the NT plants with, for example, cucumber mosaic virus. Seeds harvested from NT pollen recipients containing fluorescent embryos will indicate a cross-pollination event.

Paternity experiments using naturally occurring DNA sequences and sequence variants as markers to investigate insect-mediated pollen transfer are useful for studies of gene flow in plants.^12^^,^^13^ To investigate pollinator-mediated gene flow between plants we used transgenic tomato plants of the Moneymaker variety carrying the coding sequence for GFP under the control of the constitutive cauliflower mosaic virus 35S promoter.^14^ Inheritance of this artificial marker gene was scorable visually because it conferred fluorescence on seed tissue as well as most other plant tissues, and it allows investigation of pollen transfer using plants that are genetically identical except for the possession, or not, of the *GFP* transgene (Figure 1).

Expression of the *GFP* transgene in this tomato line (GFP#6) induces no significant changes in carbon metabolism^14^ and is therefore unlikely to have altered bee-perceivable cues such as changes as emission of volatile organic compounds or floral pigments. Nevertheless, we checked that genetic modification had not affected seed yield or influenced in some way the buzz-pollination behavior of bumblebees. Fifty-plant arrays comprising 25*GFP*-transgenic and 25 non-transgenic plants arranged alternately were set up in a glasshouse flight arena with a bumblebee nest placed centrally (Figure S1). To confirm that *GFP* transgene expression did not influence bumblebee buzz-pollination behavior, we measured the time bumblebees spent sonicating flowers, i.e., gripping the floral cone and vibrating to release pollen (Figure S2A). Bees sonicated flowers of non-transgenic plants and *GFP*-transgenic plants for similar durations (Figure S2A). Fruits arising from sonicated flowers (i.e., visited by bumblebees) and fruits that developed from unvisited flowers were harvested and the seeds collected and counted. For fruit produced by unvisited flowers, the number of seeds per fruit was similar for non-transgenic and *GFP*-transgenic plants (Figure S2B). As expected from previous investigations,^11^^,^^15^ buzz-pollination significantly increased seed production, and the enhancement of self-pollination was statistically indistinguishable between non-transgenic and *GFP*-transgenic plants (Figure S2B). Thus, constitutive expression of the *GFP* transgene did not affect any tomato phenotype influencing reproduction or cues affecting bumblebee pollination behavior.

### Bumblebee mediated pollen transfer was enhanced from virus-infected tomato plants

**Figure 2:**
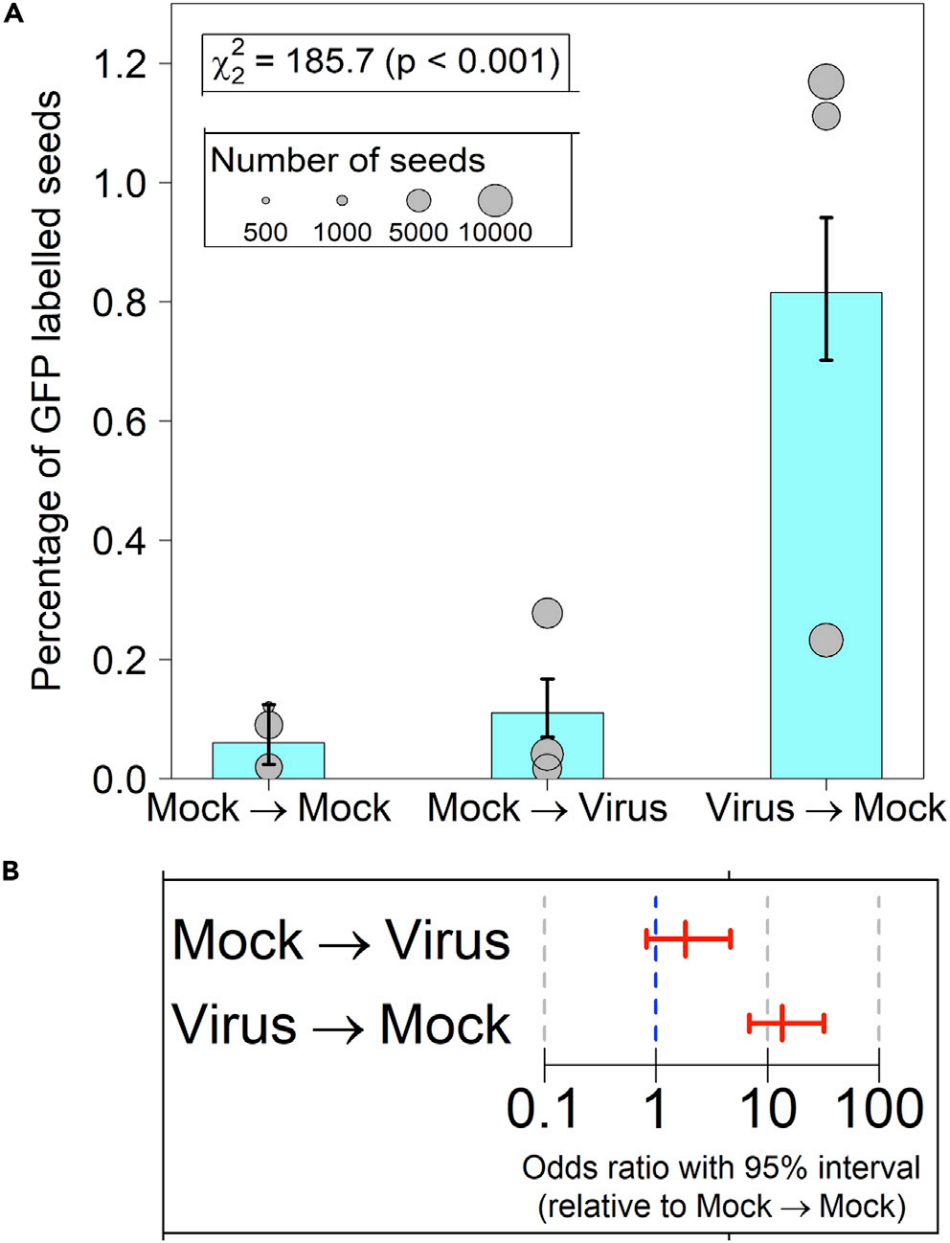
Bumblebee mediated cross-pollination is enhanced from CMV-infected tomato plants (A) The percentage of seeds from non-transgenic ‘pollen recipient’ plants showing GFP fluorescence was used as a measure of pollen transfer from healthy GFP-expressing plants to healthy non-transgenic plants (Mock→Mock), from healthy GFP-expressing plants to CMV-infected NT plants (Mock→Virus), and CMV-infected GFP-expressing plants to healthy NT plants (Virus →Mock). The inheritance of the GFP transgene differed between treatments ($\chi^{2}\left(2\right)=185.7;p<0.001)$. The proportion of pollen movement from Virus→Mock was different from both Mock→Mock and Mock→Virus, but the proportions in Mock→Mock and Mock→Virus did not significantly differ from each other. Mean values and SEM are shown. The three datapoints for each replica experiment are shown as circles, the size of which relates to the total number of seeds harvested from the ‘pollen-recipient’ plants in each experiment. (B) Summary of cross-pollination data to determine the likelihood of transmission from healthy and virus-infected pollen donor plants. The *x*-axis shows the range of the 95% confidence interval of the probability of transmission of the GFP-transgene from mock-treated to infected plants (Mock→Virus), and vice-versa (Virus→Mock), using the mock-to-mock transmission rate as a baseline. Fitted 95% confidence intervals on odds ratios relative to mock-mock: $0.82<\exp\left(\alpha_{MV}\right)<4.63$ (Mock→Virus); $6.89<\exp\left(\alpha_{VM}\right)<31.9($ Virus→Mock).

Six weeks after either mock or CMV inoculation, experiments using *GFP*-transgenic plants were carried out to measure the rate of pollen transfer by bumblebees from: flowers of mock-inoculated plants to flowers of other mock-inoculated plants; flowers of mock-inoculated plants to flowers of CMV-infected plants, and flowers of CMV-infected plants to those of mock-inoculated plants (Figure 1). Fruit was collected from non-transgenic plants and seeds examined for expression of GFP using a low magnification epi-fluorescence microscope. Seeds resulting from self-pollination were not fluorescent, but seeds resulting from cross-pollination fluoresced because of the presence of GFP in the embryo, i.e., demonstrating that fertilization of the seed had resulted from bumblebee-mediated transfer of *GFP*-transgenic pollen to the flower of a non-transgenic plant (Figures 1 andS3). The cross-pollination rate between non-infected plants, that is, the basal rate of cross-pollination in the absence of virus infection, ranged from 0.01 to 0.1% in three independent experiments (Figure 2A; Table S1). This low basal rate of cross-pollination is consistent with the predominantly autogamous (self-fertilizing) reproduction of tomato, and with previous estimates of natural cross-pollination in the field. For example, Groenewegen et al.^16^ used a transcription factor gene controlling anthocyanin biosynthesis^17^ as a paternal marker to detect crosses with a tomato line homozygous for the mutant allele and scored seedlings for production of anthocyanin. This approach yielded a mean value for the cross-pollination rate of 0.0721% when pollinators were present,^16^ which is commensurate with our estimated rate of cross-pollination (Figure 2A; Table S1).

The rates of bee-mediated pollen transfer from mock-inoculated to CMV-infected plants and from CMV-infected plants were compared to the basal cross-pollination rate (Figure 2). Analysis by either binomial logistic regression (Figure 2A) or probability (Figure 2B) showed the proportion of pollen transferred from mock-inoculated plants to CMV-infected plants was not significantly elevated relative to the basal cross-pollination rate between healthy plants. However, pollen transfer from CMV-infected plants to mock plants was enhanced by an order of magnitude, relative to the basal cross-pollination rate (Figure 2A). This remarkable observation indicates that CMV infection leads to a markedly increased probability of transfer of pollen from infected plants to non-infected plants, whereas having no significant impact on pollen movement in the opposite direction.

**Figure 3:**
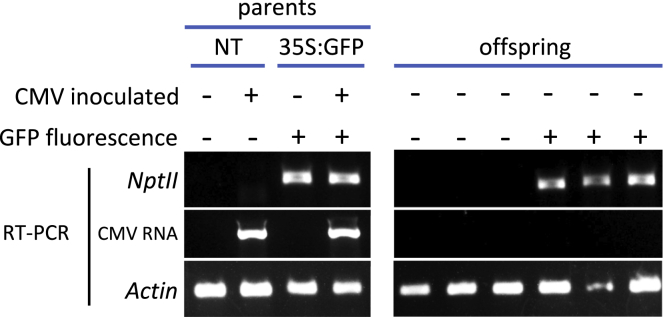
Confirmation by RT-PCR that seeds showing GFP fluorescence were the result of a cross-pollination event The right panel shows that *NptII* expression (demonstrated by RT-PCR with appropriate primers, labeled NptII) is detected in parent plants that are transgenic for the 35S:GFP construct but not in non-transgenic parents (NT). The left panel shows that some seeds (offspring) from non-transgenic parents occasionally showed GFP fluorescence (+) and also expressed *NptII* and that no offspring were infected with CMV (established by RT-PCR using appropriate primers, labeled CMV). This confirmed that fluorescent seeds were the result of cross-pollination. RT-PCR was performed on RNA extracted from parent plants and germinated offspring. cDNA was generated using random primers and subsequently used in PCR reactions using appropriate primers (Table S3) for NptII and CMV. A control PCR was also carried out on the same cDNA for tomato actin (Actin) as a quality control measure.

All GFP fluorescent seeds were viable. Seedling leaf tissue was used to carry out reverse transcription-coupled polymerase chain reactions (RT-PCRs) to detect expression of the transcript of the *neomycin phosphotransferase II* (*NPTII*) transgene. *NPTII* (conferring kanamycin resistance) is present in the transfer DNA containing the *GFP* marker gene.^14^^,^^18^^,^^19^ RT-PCR showed that seeds showing GFP fluorescence also expressed *NPTII*, confirming that pollen from a *GFP*-transgenic tomato plant had been transferred by a bumblebee to the stigma of a non-transgenic plant (Figure 3). Seeds resulting from cross-pollination germinated successfully, showing that neither cross-pollination nor CMV infection of the paternal parent plant affected the viability of the resulting seed. We found that progeny from crosses were not infected with CMV (Figure 3). This is consistent with the previous literature on CMV, i.e., that this virus is not known to be transmitted via tomato pollen, and that bumblebees are not CMV vectors.^20^^,^^21^ A selection of self-pollinated seeds from fruits harvested from healthy and CMV-infected plants had similarly high germination rates also indicating that seeds from virus-infected plants were as viable as those from healthy plants (Figure S4).

### Bumblebees spent less time interacting with flowers on CMV-infected plants

**Figure 4:**
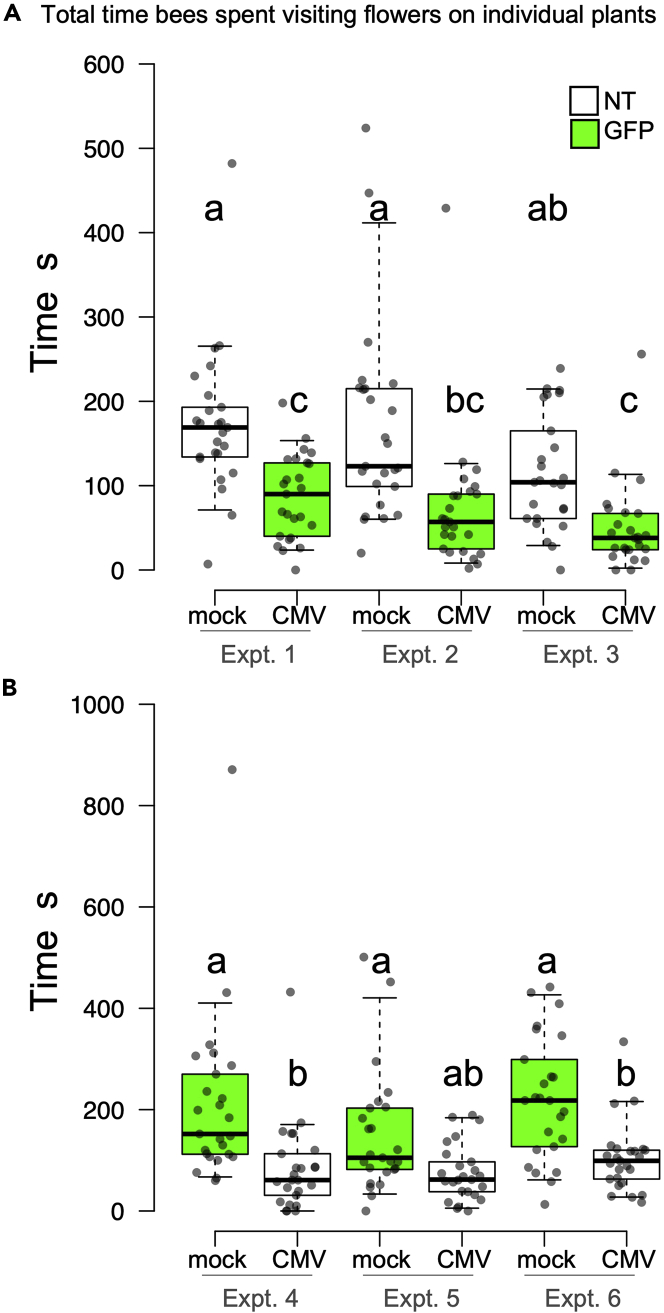
Bumblebees spend less time buzz-pollinating virus-infected plants compared to healthy plants Panel A and B show the aggregate time that bees spent interacting with the flowers on individuals in a 50 plant (5 X 10) array of alternating mock-inoculated and CMV-infected plants. Panel A shows how long plants and bees interacted in an array where the mock-inoculated plants were non-transgenic (NT) and the virus-infected plants were transgenic for 35S:GFP (GFP, green boxes). Panel B shows how long plants and bees interacted in the reciprocal experiment where mock-inoculated plants were transgenic for 35S:GFP whereas CMV-infected plants were non-transgenic. Boxplots were generated using ‘BoxPlotR: a web-tool for generation of boxplots’ available at http://shiny.chemgrid.org/boxplotr/.^22^ Center lines show the medians; box limits indicate the 25th and 75th percentiles as determined by R software; whiskers extend to 5th and 95th percentiles; data points are plotted as dots. n = 25 plants. Three independent biological replicates are shown, i.e. each experiment is carried out with a new set of plants and naive bumblebees.

To understand how the bias for bumblebee-mediated pollen transfer from infected to non-infected plants occurs, we first examined if bumblebee foraging behavior differed between visits to CMV-infected and mock-inoculated plants. Previous work suggested that bumblebees may spend more time foraging on flowers from CMV-infected plants.^11^ However, using bee tracker software to time flower visits of bees over the course of a 2-3h pollination experiment and by producing a larger dataset, we found that bees consistently spent half as long visiting flowers on infected plants than they did visiting flowers on healthy plants (Figure 4). This was largely owing to bees paying fewer visits to virus-infected plants (Figure S5).

**Figure 5:**
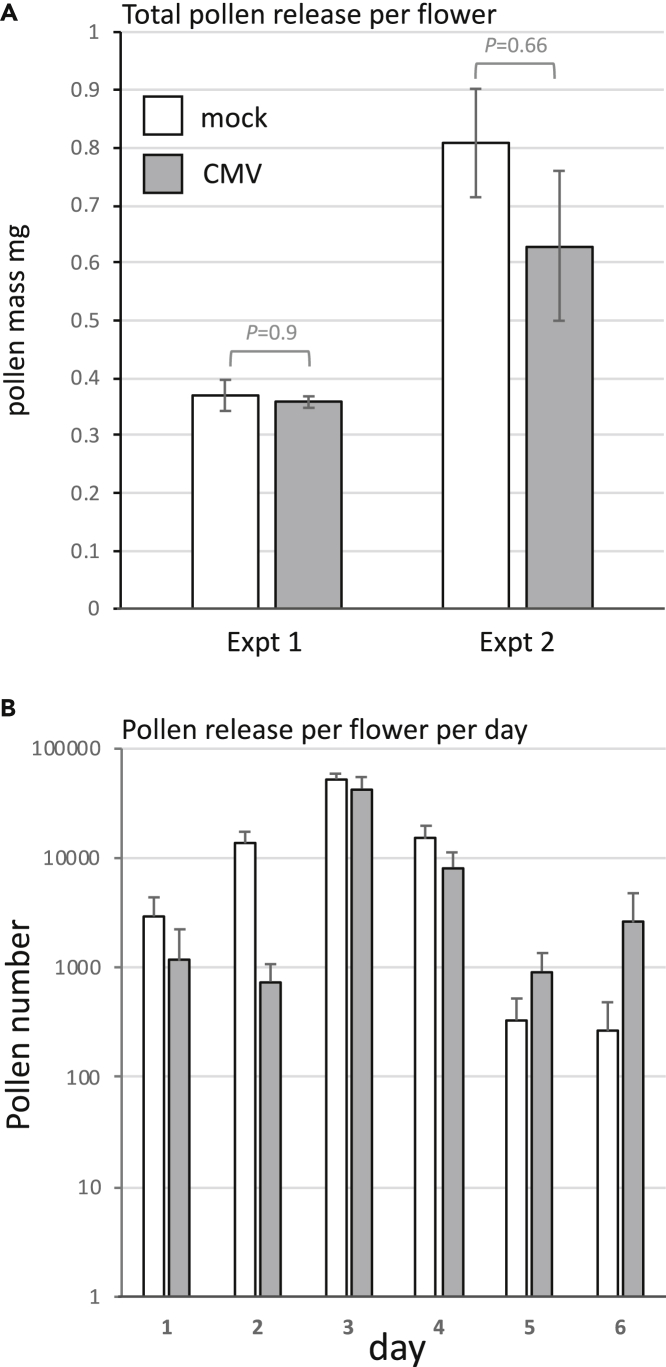
Pollen release from anthers of healthy and CMV-infected tomato plants (A) Total pollen was collected from ripe flowers by sonication with an electric toothbrush over a period of days (until no more pollen was seen to be released) and weighed using a microbalance. Mean values and SEM are shown (n = 4 plants, >28 flowers) for two experiments. Analysis by Student’s *t*test showed no statistical difference between the mass of released pollen from healthy or CMV infected plants. (B) Fully developed flowers were sonicated with an electronic toothbrush for 5 s once a day for six days. Released pollen was counted. Mean values and SEM are shown (n>8). Substantial release of pollen (*c*. 10^4^ grains or more) occurred for 3 days in healthy plants, whereas the window for pollen release in CMV-infected plants was limited to two days.

Could the amount of pollen released from virus-infected plants as a reward to foraging bumblebees be lower than that from non-infected plants and therefore account for the bees’ fewer visits to virus-infected plants? Although the pollen of CMV-infected plants is as viable as that from non-infected plants,^11^ we wondered if pollen maturation or the ease with which it would be released by sonication would be affected on CMV-infected plants. Our rationale for this hypothesis was that the CMV 2b protein is known to inhibit expression of *c*. 90% of plant genes regulated by the phytohormone jasmonic acid (JA)^23^ and because, among other things, JA is a key factor controlling pollen development in many plants, although in tomato it appears to be less critical.^24^ Pollen was released by mechanical sonication of flowers and weighed using a microbalance. No significant difference in total pollen mass per flower was detected (Figure 5A). But although CMV infection of tomato did not decrease the overall quantity of pollen produced, it did affect the timing of peak pollen availability (Figure 5B). We found that although flowers of non-infected plants released peak amounts of pollen over three days (≥10^4^ grains per 5 s vibration), flowers of CMV-infected plants released most of their pollen over a two-day period (Figure 5B). Although it is unclear how this change in timing would explain the bias for pollen transport from CMV-infected to uninfected plants, it may explain why, overall, bees spend less time interacting with flowers on CMV-infected plants (Figures 5 andS5).

### Bumblebees were more likely to visit the flowers of non-infected plants after foraging on flowers of CMV-infected plants

Based on evidence from a combination of paternity experiments and modeling of bee-mediated pollination of plant species, including *Brassica rapa* and *Medicago sativa*, pollinator residence has been proposed as being inversely proportional to gene flow mediated by by cross-pollination.^25^^,^^26^ That is, gene flow via cross-pollination is inhibited if pollinators remain on the same plant or if they subsequently visit only plants of the same genotype. In contrast, gene flow is favored when pollinators migrate to forage on plants of the same species but of differing genotype. The greatest chance of a cross-pollination event occurring will be when two flowers are visited in immediate succession.^25^^,^^26^

**Figure 6:**
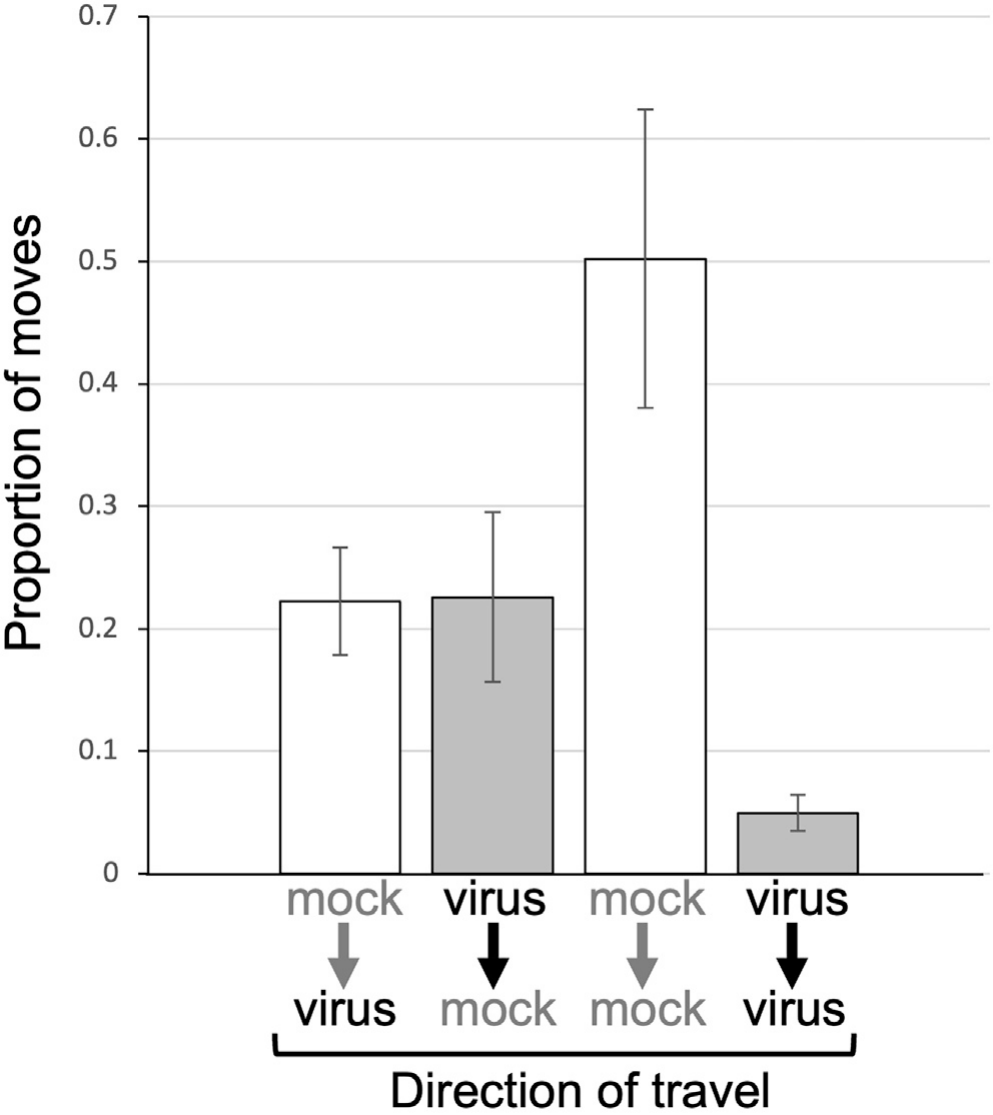
Bumblebee migration between flowers on CMV-infected tomato plants is significantly lower than between healthy plants The movement pattern of individual bees between flowers of virus-infected and mock-inoculated plants in a 3 X 10 array was recorded. Four bee ‘destinations’ were possible, in that bees could move: from flowers on mock-inoculated to CMV-infected plants (mock→virus) and vice-versa (virus→mock); between flowers on different mock-inoculated (mock→mock) or different CMV-infected plants (virus→virus). Comparison of these choices indicated that bee choices were biased [chi-square (χ^2^) goodness of fit test p < 10^−5^] and that the rarest move was between flowers on virus-infected plants. The moves presented (as a proportion) are the mean of 3 biological replicates. The number of bee moves in each replicate was 31, 34 and 39). Mean values and SEM are shown.

We set up experiments to track bumblebee movement between flowers of mock-inoculated and CMV-infected plants to find out if the cross-pollination bias (Figure 2) was explainable by patterns of pollinator foraging behavior, i.e., did the likelihood of pollinator residence differ for infected versus non-infected plants? The patterns of movement between flowers for individual bumblebees were recorded in arrays of 3 x 10 plants. These arrays comprised alternating CMV-infected and mock-inoculated tomato plants (Figure S6). A bumblebee under these conditions, following its initial visit to a flower, had two foraging choices available. First, it might move to a plant of the same treatment group (from a mock-inoculated plant to another mock-inoculated plant, or from a CMV-infected plant to another CMV-infected plant), and this would be ‘residence’ in the modeling terminology of Cresswell et al.^25^ Alternatively, the bumblebee might move to a plant of the other treatment group, i.e., from a CMV-infected plant to a mock-inoculated plant or from a mock-inoculated plant to a CMV-infected plant. If bumblebees move in an unbiased fashion from flower to flower, an even split between choices would have been expected. Initial choices of individual bumblebees for their initial foraging bout were unbiased between CMV-infected and mock-inoculated plants. However, it was found consistently that the rarest subsequent move by a bumblebee was from a virus-infected plant to another virus-infected plant, and that bumblebees that alighted first on flowers of mock-inoculated plants showed the highest degree of residency, that is, choosing to visit another mock-inoculated plant (Figure 6, Table S2). Thus, CMV infection of tomato affected bumblebee-flower interactions in that it was more likely that a bumblebee would visit a flower on a mock-inoculated plant after buzz-pollinating a flower on an infected plant. In the light of previous analyses of gene flow in plant-pollinator systems,^25^^,^^26^ this provides a mechanism to explain why transfer of pollen carrying a *GFP* marker gene is more probable when the donor (i.e., the male parent) is infected with CMV (Figure 2).

### Conclusions

Pollen from flowers of CMV-infected plants was more likely to be transferred by bumblebees to the stigmas of non-infected plants. This bias may be caused by distasteful substances accumulating in the pollen or differences in, for example, the tactile cues of the flower, or by other factors to be investigated in future work. These results may explain the increased success of infected plants as male parents in cross-pollination (Figure 2), although they do not throw light on the mechanism by which female reproduction (seed production) of CMV-infected plants is rescued by bumblebee-mediated self-pollination.^11^ Olfactometry experiments have shown that bumblebees are attracted to the VOCs emitted by virus-infected plants.^10^^,^^11^ Under the conditions used in the present study it does not appear that CMV-induced changes in VOC emission influenced the initial choices of bumblebees to settle on infected or non-infected plants. We can speculate that VOCs may play some role in enhancing bee visitation to CMV-infected plants, for example, by compensating for the decreased salience of infected plants, which are markedly shorter than mock-inoculated plants (Figure S7) or, paradoxically given their attractiveness in olfactometry experiments, VOCs may provide a cue for bumblebees that had already visited an infected plant to avoid visiting another infected plant (Figures 4 and 6). Our main conclusion is that gene flow via pollen is increased in tomato when plants are infected with CMV. This increased male reproductive success, combined with the rescue of seed production for infected plants when pollinators are present,^11^ will counteract the negative effects of viral infection on reproductive fitness, thus providing a ‘payback’ to susceptible host plants, which is not available to resistant hosts. As mentioned earlier, under natural conditions gene flow may aid evolution, but it can also counter genetic drift in an interbreeding population and promote genetic homogeneity.^2^^,^^3^^,^^4^^,^^5^ We suspect that virus-induced gene flow will tend to favor homogenization. Thus, if viruses enhance the paternity of wild plants under natural conditions, a by-product of increased gene flow from infected plants would be increased spread of host genes from the infected plant, among which will be those encoding factors that support virus infection. This idea is consistent with a hypothesis put forward by Groen et al.^11^ that for wild plants under natural conditions such a payback would promote the dissemination of alleles for susceptibility and would counteract the selective advantage of alleles for resistance. Once established, such a situation would be advantageous both for the virus and its susceptible plant hosts.

### Limitations of the study

Our study was carried out in a glasshouse and although this approach provides robust data on the influence of plant virus infection on bumblebee-mediated cross-pollination, bumblebee interactions will be more complex in the wild environment, where environmental and other biotic factors may influence pollinator-plant interactions.

## STAR★Methods

### Key resources table

**Table:** 

REAGENT or RESOURCE	SOURCE	IDENTIFIER
**Bacterial and virus strains**		

CMV isolate PV0187	German Collection of Microorganisms and Cell Cultures	DSMZ, www.dsmz.de

**Experimental models: Organisms/strains**		

*Solanum lycopersicum* L. cv. Moneymaker	Kings Seeds,Essex, UK https://www.kingsseeds.com/products/vegetable-seeds/tomato	Product code 14,810
*Solanum lycopersicum* L. cv. Moneymaker tomato line GFP#6	Prof. Fernando Carrari, Instituto Nacional deTecnología Agropecuaria, Buenos Aires, Argentina	Quadrana et al., 2011
*Bombus terrestris* L.	Koppert, Berkel en Roderijs, The Netherlands.	https://www.koppert.com/natupol/

**Oligonucleotides**		

CTCGAGCAGTGTTTCCCAGT	This paper	*S. lycopersicum Actin* Forward
GGTGCCTCAGTCAGGAGAAC	This paper	*S. lycopersicum Actin* Reverse
AATCGGCTGCTCTGATGC	This paper	Neomycin phosphotransferase II Forward
TTTCTCGGCAGGAGCAAG	This paper	Neomycin phosphotransferase II Reverse
GTGGAACGGGTTGTCCATCCAGCT	Groen et al., 2015	Cucumber mosaic virus 3′UTR Forward
CACCCGTACCCTGAAACTAGCACG	Groen et al., 2015	Cucumber mosaic virus 3′UTR Reverse

**Software and algorithms**		

BeeTracker	This paper.	https://github.com/elderfd/BeeTracker

### Resource availability

#### Lead contact

Further information and requests for resources and reagents should be directed to and will be fulfilled by the lead contact, Alex M. Murphy.

#### Materials availability

This study did not generate new unique reagents.

### Experimental model and subjectdetails

Tomato seeds (*S. lycopersicum* L. cv. Moneymaker) were obtained from Kings Seeds (Essex, UK). Seeds for transgenic tomato line GFP#6 (also in the Moneymaker genetic background) expressing the *GFP* gene was kindly provided by Prof. Fernando Carrari (Instituto Nacional deTecnología Agropecuaria, Buenos Aires, Argentina).^14^ Seeds were germinated in 9 cm diameter Petri dishes with water for seven days before transfer to individual P24 tray inserts with cells 50 × 48 mm in size (Desch Plantpak, Mundon Maldon, Essex, UK) containing Levington M3 compost (Fisons Plc., Ipswich, UK). Tomato seeds were germinated and seedlings initially grown under a photoperiod of 16 h light/8 h dark at 22°C ± 1°C, 60% relative humidity and a light intensity of 200 μE m^−2^ s^−1^ in a custom-built walk-in growth chamber with an automated watering system on a 36 h cycle (Conviron, MB, Canada) at the Plant Growth Facility, Botanic Garden, University of Cambridge, UK.

CMV isolate PV0187 (Subgroup IA) was obtained from the German Collection of Microorganisms and Cell Cultures (DSMZ, www.dsmz.de).^11^ Virions were purified from *Nicotiana benthamiana* 3 weeks post-inoculation and stored for up to 3monthsat 4°C.^27^ Seedlings were mechanically inoculated on both fully emerged cotyledons after one week of growth by dusting with Carborundum (silicon carbide) powder and gently rubbing 10 μL of purified CMV virions (100 μg mL^−1^ suspension in sterile water) with a frosted glass microscope slide over the cotyledon surface. Mock-inoculated plants underwent the same procedure with sterile water.

At two weeks of age, inoculated and mock-inoculated plants were transplanted into 12 × 12 × 20 cm pots and grown for another two weeks under controlled growth conditions before transfer to the University of Cambridge Botanic Garden glasshouse. Plants were maintained at 15–25°C, and Lucalux LU 400 W/PSL lights were automatically activated between the hours of 0400 and 2000 when daylight levels fell below 150 W/m^2^. Humidity was approximately 55%. Tomato plants were placed randomly within the glasshouse space available (to minimize the potential environmental gradient effects such as light or humidity) and after beginning to flower (3-6 weeks later) used in pollination experiments.

### Method details

#### Cross-pollination experiments

Fifty flowering tomato plants were arranged in a 5 x 10 array in a flight arena bounded by insect-proof netting (320 × 440 × 210 cm, W x L x H; JoTech-Insectopia Ltd., Austrey, UK; Figures S1A–S1C). A boxed colony of bumblebees (*B. terrestris* L. sourced from Koppert, Berkel en Roderijs, The Netherlands) was introduced into the center of the flight arena and bees were allowed free access to flowers and their colony. To measure pollen transmission from virus-infected to mock-inoculated plants, CMV-infected *GFP*-transgenic tomato plants and mock-inoculated non-transgenic tomato plants were arranged alternately in the plant array within the flight arena (Figure S1D). To measure pollen transmission from mock-inoculated to virus-infected plants, the reciprocal experiment was carried out, i.e. mock-inoculated *GFP*-transgenic and CMV-infected non-transgenic tomato plants were used. Pollen transmission rates were also measured between mock-inoculated *GFP*-transgenic and non-transgenic tomato plants. When bumblebees had visited all the plants at least once, the experiment was concluded and all buzz-pollinated flowers (identified from characteristic wounding marks left by bees after buzz-pollination; Figures S1D–S1F) were labeled with a jeweler’s tag (Figure S1G). Fruits arising from buzz-pollinated and non-pollinated flowers were allowed to develop on these plants and harvested (8-12 weeks later). The seed from individual fruit was collected by rinsing through a sieve.

To determine seed paternity, the presence of marker genes in tomato seeds was ascertained by epi-fluorescent microscopy (M165 FC Fluorescence Dissecting Stereomicroscope equipped with a GFP filter, Leica Microsystems, Milton Keynes, UK) for the GFP protein to ascertain the cross-pollination rate. GFP fluorescing progeny seed harvested from non-transformed tomato plants were indicative of a cross-pollination event (Figure S3). These seeds were germinated, and RNA was extracted from cotyledons using Norgen Total RNA Purification Plus Kit (Norgen Biotek, Thorold, ON, Canada) and used in RT-PCR for *NPTII* (kanamycin resistance gene) as further confirmation that these seeds were progeny of a *GFP*-transgenic pollen donor and a non-transgenic pollen recipient (primers used: Table S3). Experiments to measure the rate of pollen transfer from GFP-expressing CMV-infected plants to NT healthy plants were carried out three times. The reciprocal experiments (transfer of pollen from GFP-expressing healthy plants to NT CMV-infected plants) and the control experiments (transfer of pollen from GFP expressing healthy plants to NT healthy plants) were also carried out three times.

#### Mechanical release and collection of pollen

As described by Groen and colleagues,^11^ artificial buzz-pollination was carried out using an electrically actuated toothbrush (‘Oral-B’: Proctor and Gamble, Cincinnati, USA). The brush was covered with a finger from a disposable glove, which was changed between flowers.

To measure mass per flower, pollen was collected by holding a microfuge tube around the anther cone while sonicating the back of the flower with the toothbrush. Pollen from all open flowers on a plant was collected into a single weighed microfuge tube every day from when flowers first opened with petals curled right back, or ‘stage 6’ flower development, according to Dobritzsch et al.^28^ to when they collapsed. The sum of all pollen yields from a single plant was divided by the number of flowers sonicated, to give a mean mass per flower.

To evaluate the pollen yield per day from open (stage 6) tomato flowers, pollen released from 5 s of sonication was collected into a single microfuge tube every day from when flowers first opened to when they collapsed. Pollen released from each flower per day was suspended in 200 μL of water and counted in technical triplicates using a cell-counting chamber under a microscope.

### Quantification and statistical analysis

Data from cross pollination experiments shown in Figure 3 were analyzed using binomial logistic regression. Data from the *i*^th^ experiment consists of a count of the number of GFP labeled seeds, *s*_*i*_, together with a count of the total number of seeds collected in the experiment as a whole, *N*_*i*_. We fitted the following model to these data, modeling $p_{i}$ the probability in the *i*^th^ experiment of the pollen donor being a GFP labeled plant

$$s_{i}=Bin\left(N_{i},p_{i}\right)\text{,}$$

$$\log\left(\frac{p_{i}}{1-p_{i}}\right)=\alpha_{MM}+\alpha_{MV}\cdot \chi_{MV}\left(i\right)+\alpha_{VM}\cdot \chi_{VM}\left(i\right)\text{,}$$

in which$\alpha_{MM}$ is the fitted logarithmic odds ratio in the mock-mock experiments, $\alpha_{MV}$ and $\alpha_{MV}$ are treatment effects for mock-virus and virus-mock experiments, respectively, and the indicator function $\chi_{MV}\left(i\right)$ and $\chi_{VM}\left(i\right)$ reflect the treatment in the *i*^th^ experiment. Statistical differences between any pair of treatments was tested by comparing the fit of this model with the simpler nested model in which$\alpha_{MV}=\alpha_{VM}=0$ by way of a likelihood ratio test. Differences between individual treatments were then assessed by checking whether the 95% confidence intervals on the fitted treatment effects $\alpha_{MV}$ and $\alpha_{VM}$ overlapped 0 (and/or each other).

### Additional resources

#### ‘BeeTracker’

BeeTracker is a small GUI (graphical user interface) program, developed with C++ and Qt, which eases the tracking of pollinator visits to plants on a small scale (for example in behavioral arenas, cages or glasshouses), particularly when tracking the movements of multiple pollinators. BeeTracker shows a grid of configurable size, which the user matches to the experiment layout. The cells of the grid are clicked to start the timing of visit, and clicked again to indicate the visit has ended. Multiple visits can be tracked at a single location simultaneously. When the program is closed (at completion of experiment), data is automatically saved as a file containing all the recorded visits in tab-delimited columns for analysis. Access to BeeTracker code is at https://github.com/elderfd/BeeTracker.

## Data Availability

Raw data used in this study is available in the supplemental information. All original code has been deposited at GitHub. The DOI has been listed in the key resources table. Any additional information required to reanalyze the data reported in this paper is available from the lead contactupon request.
