# Supplementary material for: Biased pollen transfer by bumblebees favors the paternity of virus-infected plants in cross-pollination
Source: iScience. 2023 Feb 24;26(3):106116. doi: 10.1016/j.isci.2023.106116 (PMC10040881; doi:10.1016/j.isci.2023.106116)
Supplement: Document S1. Figures S1–S7 and Tables S1–S3 [file mmc1.pdf]

iScience, Volume *26*

Supplemental information

Biased pollen transfer by bumblebees favors the paternity of virus-infected plants in cross-pollination

Alex M. Murphy, Sanjie Jiang, James A.D. Elderfield, Adrienne E. Pate, Chay Halliwell, Beverley J. Glover, Nik J. Cunniffe, and John P. Carr

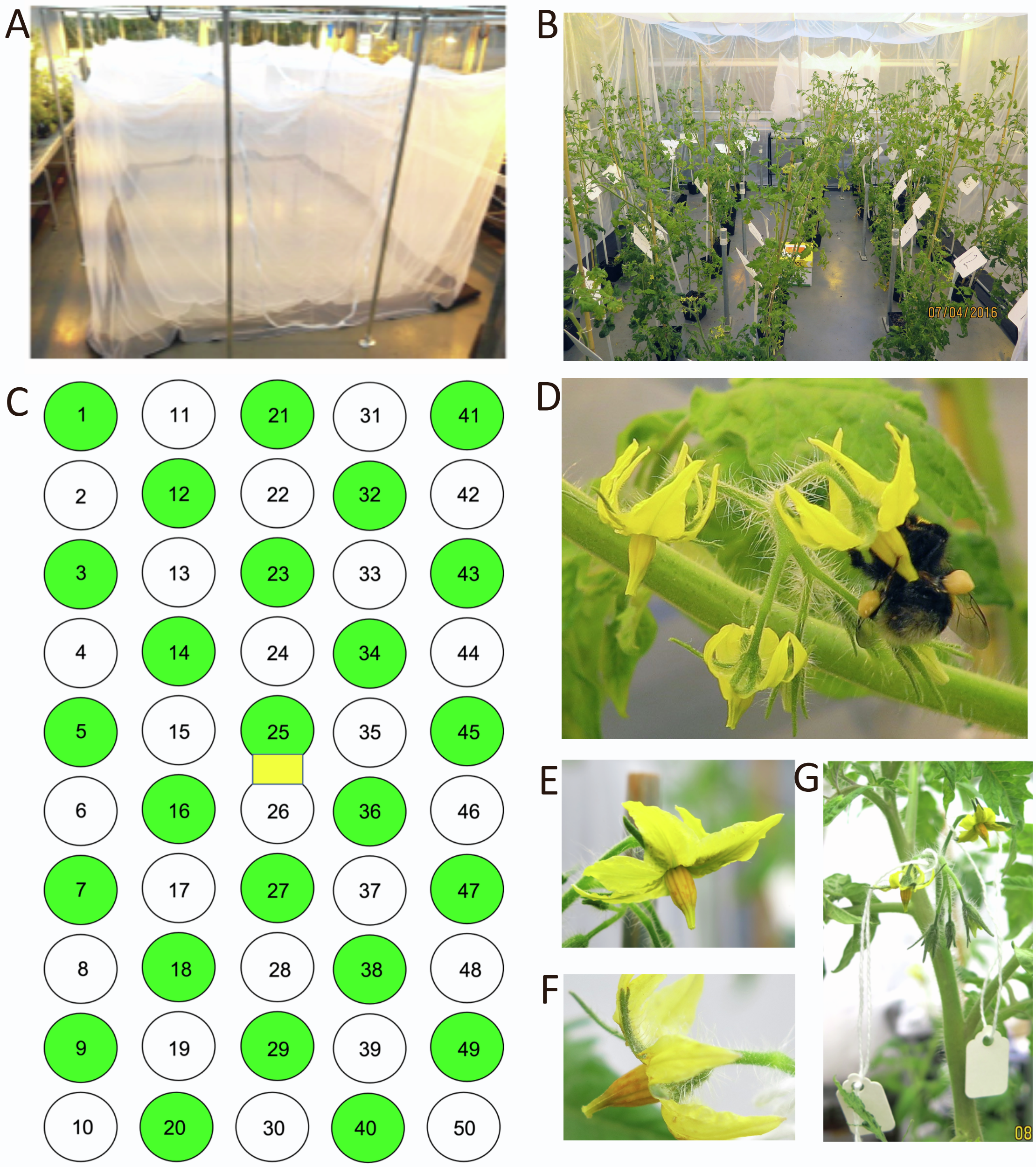

Figure S1. Details of experimental set up. Related to Figure 2.

A. Bee pollination experiments were conducted within a nylon net flight arena (320 x 440 x 210 cm, W x L x H).

B. Fifty flowering tomato plants were arranged in a 5 X10 array in an alternating pattern of transgenic plants expressing GFP and non-transformed (NT) plants (depending on experiment, NT or GFP-expressing plants were mock-inoculated or CMV-infected). A boxed colony of bumblebees was placed in the centre of the flight arena.

C. Bees were allowed free access to flowers and their colony

D. Bumblebee gripping anther cone of tomato flower during sonication

E and F. When bumblebees had visited all the plants at least once, the experiment was concluded, and all buzz-pollinated flowers were identified from characteristic wounding marks

G. Buzz-pollinated flowers were labelled with a jeweller's tag.

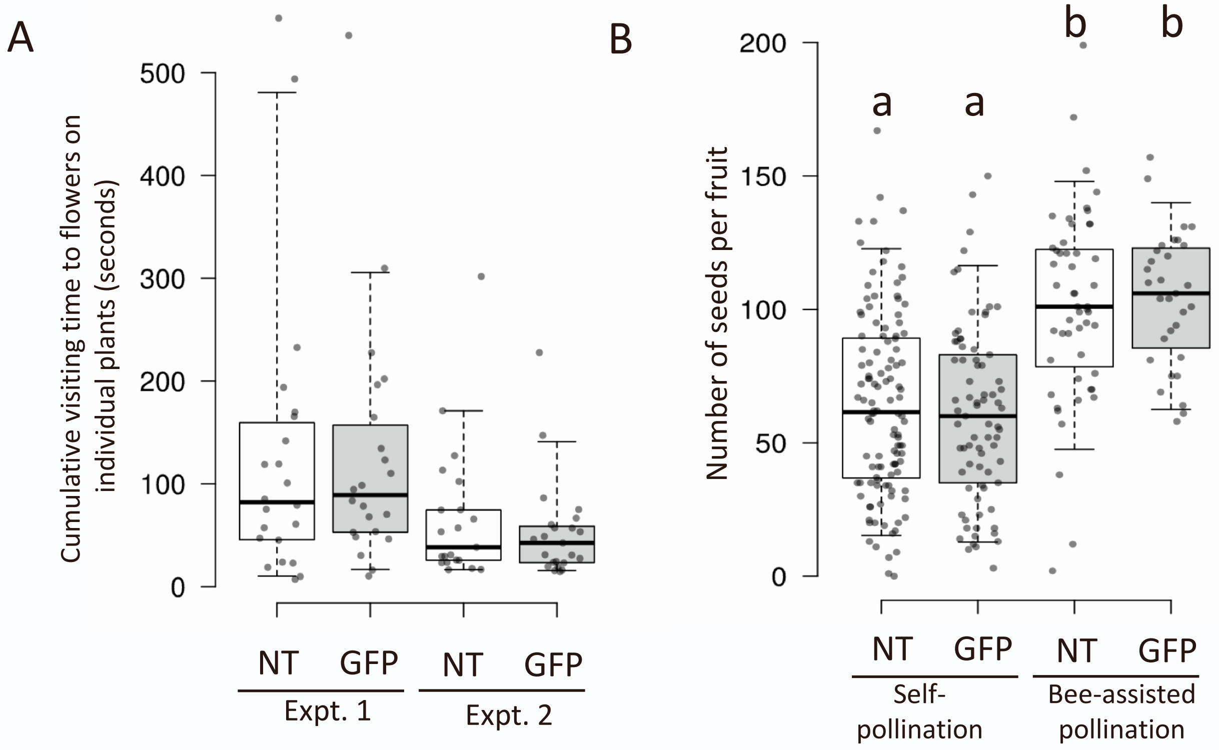

Supplementary Figure S2. Bumblebee pollination behaviour in control experiments. Related to Figure 2. A. Bumblebees spent a similar amount of time sonicating flowers on non-transformed (NT) and GFP-expressing (GFP) tomato plants. Data from two experiments is shown (Expt.1 and Expt. 2).
Centre lines show the median time that bees spent interacting with flowers, most of this time was spent sonicating the anther cone. Individual data points (grey circles) represent the cumulative time that bees spent visiting a single plant. Plants typically had 2 to 4 open flowers. N = 22, 22, 21, 23 data points (individual plants) from left to right.
B. Bumblebee sonication of flowers resulted in significantly higher seed yield that was the same for non-transgenic (NT) and transgenic 35S:GFP expressing plants. From left to right, n = 116, 77, 51, 31 tomato fruits harvested from 25 NT and 25 GFP expressing tomato plants.
Boxplots were generated using 'BoxPlotR: a web-tool for generation of box plots' available at http://shiny.chemgrid.org/boxplotr/[1]. Center lines show the medians; box limits indicate the 25th and 75th percentiles as determined by R software; whiskers extend to 5th and 95th percentiles; data points are plotted as dots.

1. Spitzer M., Wildenhain J., Rappsilber J., Tyers, M. (2014) BoxPlotR: a web tool for generation of box plots. Nat Methods 11, 121–122. https://doi.org/10.1038/nmeth.2811

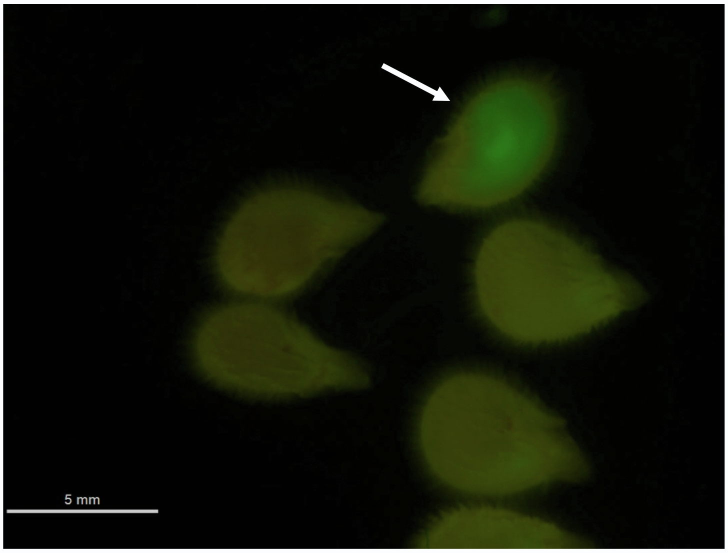

Figure S3. Appearance of a seed resulting from bee mediated transfer of pollen from a GFP-expressing plant to the stigma of a NT plant. Related to Figure 2. Arrow points to cross-pollinated seed (showing GFP fluorescence) next to seeds resulting from self-pollination (not showing GFP fluorescence).

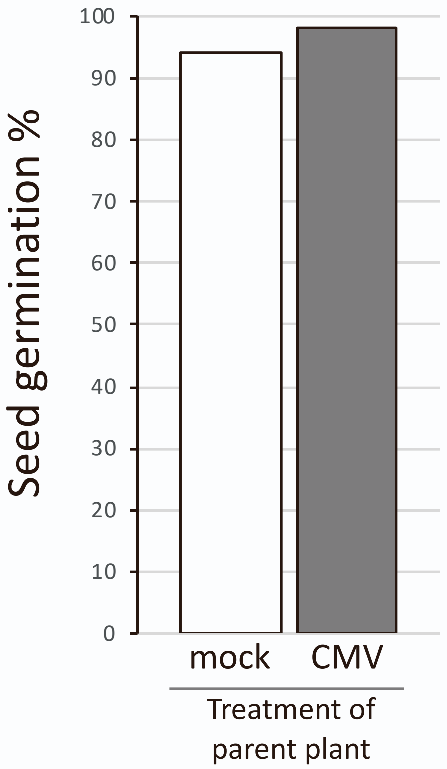

Figure S4.Germination rate of seeds from healthy and CMV-infected tomato plants was similar. Related for Figure 2. Seeds from fruit that developed from buzz-pollinated flowers were harvested and dried. Dried seed was surface sterilised and placed on MS agar and scored for germination two weeks later. In this case, seeds were from mock inoculated non-transgenic and CMV infected GFP-expressing tomato plants. n= 1551 for mock and n= 1257 for CMV.

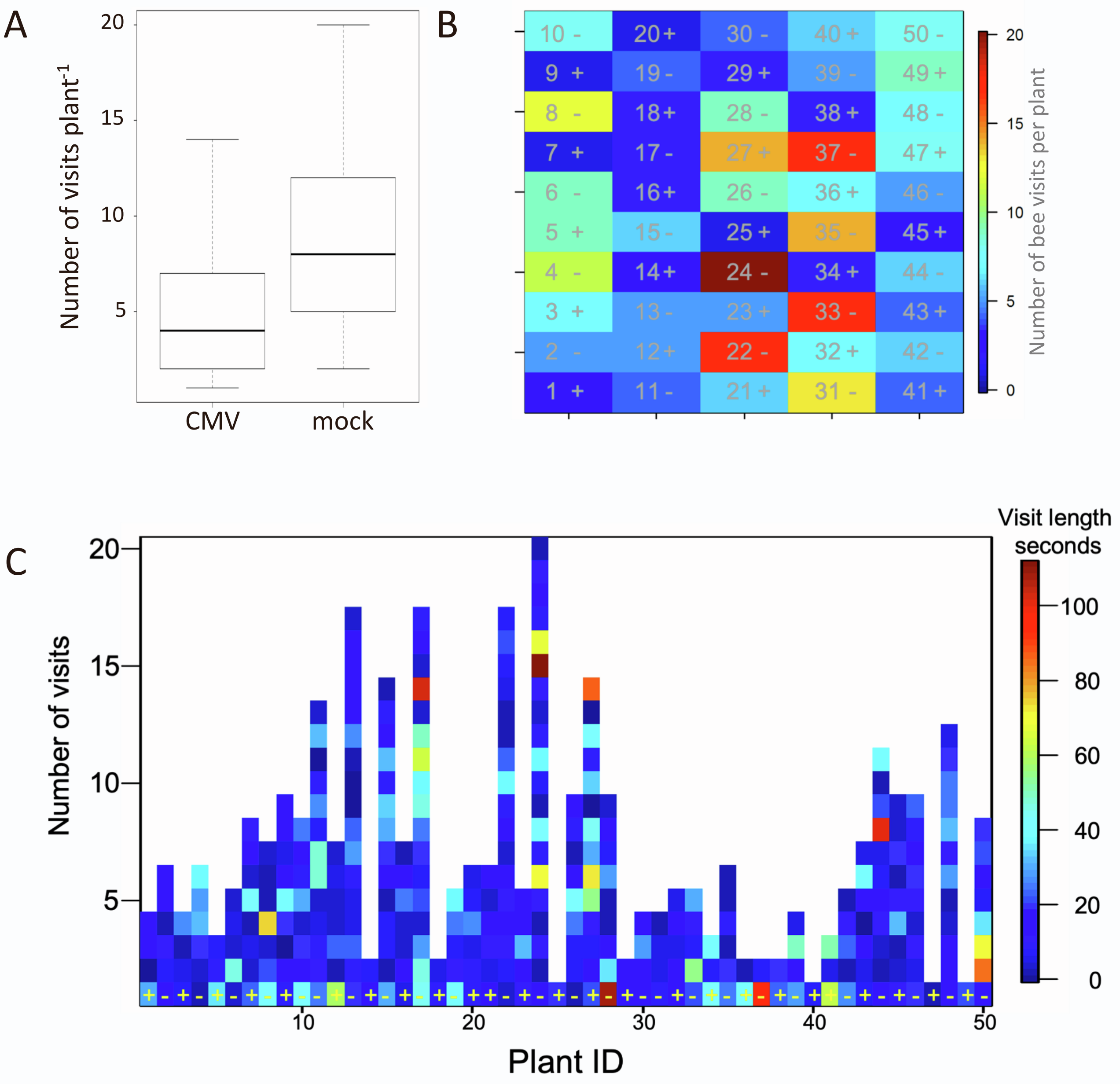

Figure S5. Bees made longer and more frequent visits to flowers on mock-inoculated plants than to virus-infected plants. Related to Figure 4.

A. Bees visited flowers on mock-inoculated plants more often than virus-infected plants (p=2x10^{-9}, Poisson regression). Boxplot center lines show the median number of visits; box limits indicate the 25th and 75th percentiles as determined by R software; whiskers extend to 5th and 95th percentiles. N = 25 plants.

B. Heatmap showing cumulative number of visits to flowers on each plant in a 5x10 array. Number =plant i.d., (+) and (-) indicate CMV-infected and mock inoculated respectively. Colour of block relates to number of visits shown in colour-bar on the left

C. Heatmap showing number and length of visits to flowers on mock-inoculated plants (-) and virus-infected plants (+). Each column represents a plant within the 5x10 array. Each block within the column represents a single bee visit the colour of which indicates the length of visit (seconds) according to the heat scale at the right of the figure.

All data compiled using the BeeTracker output file, with an example of a pollination experiment where bees had access to 25 mock-inoculated non-transgenic (mock) and 25 CMV-infected GFP-expressing (CMV) tomato plants.

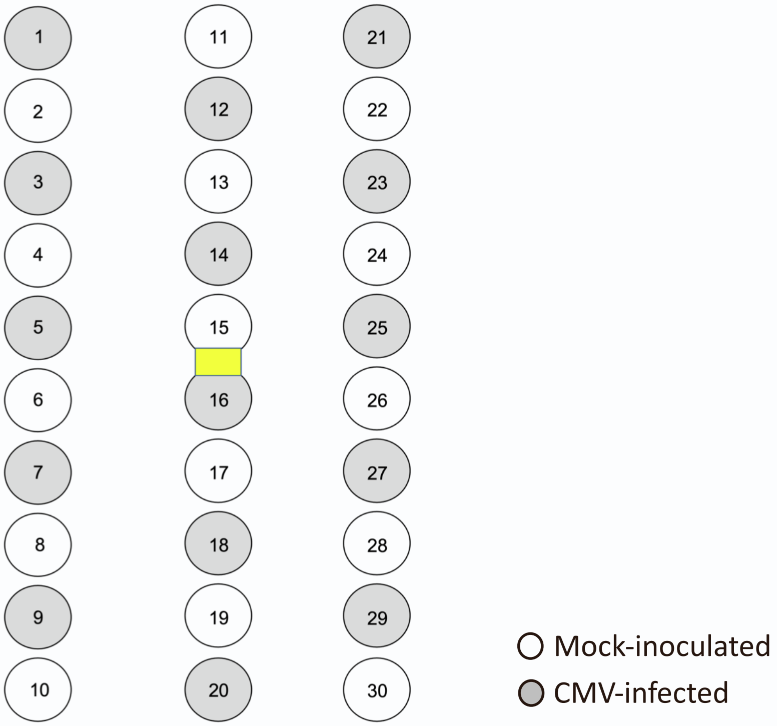

Figure S6. Arrangement of tomato plants for tracking individual bees. Related to Figure 6. Thirty flowering tomato plants were arranged in a 3 X10 array in an alternating pattern of CMV-infected and mock-inoculated plants. A boxed colony of bumblebees was placed in the centre of the flight arena. A single foraging bumblebee was released in order to track its foraging choices.

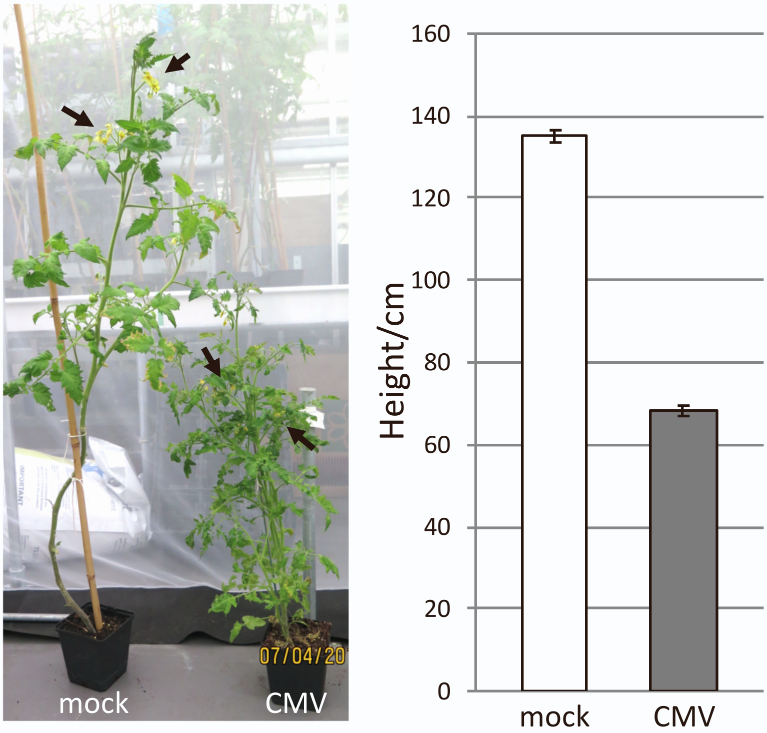

Figure S7. Characteristics of tomato plants used in bumblebee pollination experiments. Related to Figure 2 and 6. Height of mock-inoculated and CMV-infected tomato plants 6 weeks post-inoculation. CMV-infected plants are shorter compared to healthy (mock-inoculated) plants. In addition, inflorescences (arrowed) are relatively more concealed on CMV-infected plants.

Pollen donor (35S:GFP) treatment	Direction of pollen movement	Experiment number	Number of cross-pollinated seeds		
			Total number of seeds harvested from fruit that developed from buzz-pollinated flowers	Number of cross-pollinated seeds	Cross-pollination rate (%)
CMV	Virus to mock	1	8183	19	0.232%
		2	5490	61	1.110%
		3	8899	104	1.160%
mock	mock to virus	1	7385	3	0.041%
		2	6473	18	0.278%
		3	6007	1	0.017%
mock	mock to mock	1	835	1	0.119%
		2	5163	1	0.019%
		3	5561	5	0.089%

Table S1. The rate of pollen transfer by bumblebees from: flowers of virus-infected plants to flowers of mock-inoculated plants; flowers of mock-inoculated to virus-infected plants; and between flowers of mock-inoculated plants. Related to Figure 2.

Fruit was collected from non-transgenic plants and seeds examined for expression of GFP using a low magnification epi-fluorescence microscope. Seeds resulting from self-pollination were not fluorescent, and seeds resulting from cross-pollination fluoresced due to the presence of GFP in the embryo. The cross-pollination rate was calculated as the percentage of GFP-fluorescent seeds in the total number of seeds harvested from buzz-pollinated fruit from non-transgenic plants.

Expt no.	1				Expt no.	2				Expt no.	3			
Array size	3 X 10													
Bee i.d	1				Bee i.d	2				Bee i.d	3			
move	m to v	v to m	m to m	v to v	move	m to v	v to m	m to m	v to v	move	m to v	v to m	m to m	v to v
1		1			1		1			1			1	
2			1		2	1				2	1			
3	1				3				1	3		1		
4				1	4		1			4	1			
5		1			5	1				5		1		
6			1		6		1			6	1			
7			1		7	1				7		1		
8	1				8		1			8			1	
9		1			9	1				9			1	
10			1		10		1			10			1	
11	1				11			1		11	1			
12				1	12			1		12		1		
13		1			13			1		13			1	
14	1				14			1		14			1	
15		1			15	1				15			1	
16			1		16				1	16			1	
17			1		17		1			17			1	
18			1		18	1				18			1	
19			1		19		1			19			1	
20			1		20			1		20			1	
21			1		21	1				21			1	
22	1				22		1			22			1	
23		1			23	1				23			1	
24			1		24		1			24			1	
25			1		25			1		25			1	
26			1		26			1		26			1	
27	1				27			1		27	1			
28		1			28	1				28		1		
29			1		29		1			29			1	
30			1		30			1		30			1	
31			1		31			1		31			1	
					32	1				32			1	
					33		1			33			1	
					34			1		34			1	
										35	1			
										36	1			1
										37		1		
										38			1	
										39			1	

Table S2. The movement pattern of individual bees between flowers of virus-infected and mock-inoculated plants in a 3 x 10 array was recorded. Related to Figure 6. Four kinds of bee move were possible: from flowers on mock-inoculated to CMV-infected plants (m to v) and vice-versa (v to m); between flowers on different mock-inoculated (m to m) or CMV-infected plants (v to v).

Primer pair target sequence	Primer forward	Primer reverse
S. lycopersicum Actin	CTCGAGCAGTGTTTCCCAGT	GGTGCCTCAGTCAGGAGAAC
Neomycin phosphotransferase II (NPTII)	AATCGGCTGCTCTGATGC	TTTCTCGGCAGGAGCAAG
Cucumber mosaic virus Fny 3' non-translated region	GTGGAACGGGTTGTCCATCCAGCT	CACCCGTACCCTGAAACTAGCACG

Table S3. Primer sequences used in RT-PCR experiments. Related to Figure 2.
